# EZH1/2 Inhibitors Favor ILC3 Development from Human HSPC-CD34^+^ Cells

**DOI:** 10.3390/cancers13020319

**Published:** 2021-01-16

**Authors:** Laura Damele, Adriana Amaro, Alberto Serio, Silvia Luchetti, Ulrich Pfeffer, Maria Cristina Mingari, Chiara Vitale

**Affiliations:** 1 UO Immunologia IRCCS Ospedale Policlinico San Martino, 16132 Genoa, Italy; lauradamele91@gmail.com; 2 UO Epigenetica dei Tumori IRCCS Ospedale Policlinico San Martino, 16132 Genoa, Italy; adriana.amaro@hsanmartino.it (A.A.); ulrich.pfeffer@hsanmartino.it (U.P.); 3 Centro Cellule Staminali IRCCS Ospedale Policlinico San Martino, 16132 Genoa, Italy; alberto.serio@hsanmartino.it (A.S.); silvia.luchetti@hsanmartino.it (S.L.); 4 Dipartimento di Medicina Sperimentale (DIMES), Università degli Studi di Genova, 16132 Genoa, Italy

**Keywords:** NK cells, innate lymphoid cells (ILCs), innate defenses against tumors

## Abstract

**Simple Summary:**

It has been well-demonstrated that EZH1/2 enzymes are involved not only in tumor development and progression, but also in the regulation of normal hematopoiesis from CD34^+^-HSPC. Given the crucial role of NK cells in tumor immune surveillance, in this study, we investigated whether EZH1/2 inhibitors can interfere with NK cell differentiation and functional maturation. Our results suggest that EZH1/2 inhibitors push CD56^+^ precursor proliferation, skewing precursor cell lineage commitment towards ILC3. In recent years, several clinical trials on the use of EZH1/2 inhibitors against solid tumors have been carried out. Since these in vitro observations revealed possible epigenetic mechanisms involved in NK/ILC development, it is important to evaluate patient monitoring of competent NK cells repertoire in order to design appropriate therapeutic protocols.

**Abstract:**

The dysregulation of epigenetic modifications has a well-established role in the development and progression of hematological malignancies and of solid tumors. In this context, EZH1/2 inhibitors have been designed to interfere with EZH1/2 enzymes involved in histone methylation (e.g., H3K27me3), leading to tumor growth arrest or the restoration of tumor suppressor gene transcription. However, these compounds also affect normal hematopoiesis, interfering with self-renewal and differentiation of CD34^+^-Hematopoietic Stem/Progenitor Cells (HSPC), and, in turn, could modulate the generation of potential anti-tumor effector lymphocytes. Given the important role of NK cells in the immune surveillance of tumors, it would be useful to understand whether epigenetic drugs can modulate NK cell differentiation and functional maturation. CD34^+^-HSPC were cultured in the absence or in the presence of the EZH1/2 inhibitor UNC1999 and EZH2 inhibitor GSK126. Our results show that UNC1999 and GSK126 increased CD56^+^ cell proliferation compared to the control condition. However, UNC1999 and GSK 126 favored the proliferation of no-cytotoxic CD56^+^ILC3, according to the early expression of the AHR and ROR-γt transcription factors. Our results describe novel epigenetic mechanisms involved in the modulation of NK cell maturation that may provide new tools for designing NK cell-based immunotherapy.

## 1. Introduction

The dysregulation of epigenetic modifications has a well-established role in the development and progression of hematological malignancies (e.g., lymphomas, acute myeloid leukemia). In the last 10 years, a possible involvement of epigenetic regulation has been described also in the progression of solid tumors such as metastatic melanoma [1,2,3,4]. Thus, several inhibitors have been designed to interfere with enzymes that regulate DNA modifications, such as the histone methyltransferases enhancer of zeste homolog 1 and 2 (EZH1/2), the catalytic subunits of polycomb repressive complex (PRC) 1–2, which mediate trymethylation of H3K27. EZH1/2 inhibitors impaired PRC-mediated histone methylation, leading to tumor growth arrest or restoring tumor suppressor genes transcription [1,5,6,7,8]. Shields et al. found high levels of H3K27me3 and low expression of E-cadherin in non-responding melanoma patients. Moreover, EZH2 was strongly expressed in some melanoma cell lines, and the treatment with EZH2 inhibitor GSK126 induced a reduction of H3K27me3, affecting the migratory capability of melanoma cells [9].Tumor cells may exert potent immunosuppressive activity but, despite this, the immune system has been shown to play a key role in the control of both in situ disease and tumor progression [10,11,12,13]; in this context, the presence of tumor-infiltrating lymphocytes has been suggested to predict the positivity of sentinel lymph node of cutaneous melanoma [14]. Of note, in recent years a possible synergy between EZH2 inhibition and blocking the inhibitory checkpoint Cytotoxic T-Lymphocyte Associated Protein 4(CTLA-4) has been suggested, preventing melanoma expansion in resistant patients [15,16]. Accordingly, the number of clinical trials involving the use of epigenetic drugs alone or in combination with Immune Checkpoint Inhibitors (ICI) has increased and improved the prognosis of patients with metastatic disease [17,18].

Natural Killer (NK) cells belong to the Innate Lymphoid Cells (ILC) family, which is also includes the ILC1, ILC2 and ILC3 cell subsets. Each of these cell populations displays a unique functional profile, in particular, NK display an anti-tumor cytotoxicity, which they exert through the balance between activating and inhibitory receptors [19,20,21]. On the other hand, ILC1, ILC2 and ILC3 are characterized by specific patterns of cytokine production that allow them to play a relevant role in innate defenses against pathogens, epithelial tissue homeostasis and lymphoid structure organization [22,23]. NK cells can be detected in peripheral blood, and in secondary lymphoid organs such as lymph nodes (LNs), where they may play a direct role in anti-tumor response by exerting cytolytic activity, or an indirect role by promoting T cell response through DC editing [24,25]. Of note, during early development, NK cells share a common ILC precursor, but then segregate from other ILC subsets. Their development occurs primarily in the bone marrow (BM) from CD34^+^ multipotent HSPC, but they can also complete their functional maturation in lymph nodes and other tissues [26,27]. NK cell differentiation requires the expression of several transcription factors (TF), such as ID2, NFIL3, TOX1, Tbx21, ETS-1 and Eomes [27,28]. Eomes TF, in particular, is crucial for identifying late CD56^+^ precursors committed to acquiring NK cell cytotoxicity. The maturation process has been characterized ex vivo in LNs, and it requires six stages, characterized by the sequential acquisition of surface markers (including CD161, CD56, CD94/NKG2a, LFA-1, CD16, KIRs) and by functional capabilities. It is possible to detect both early CD34^+^ILC3-committed and CD34^+^ NK-committed cells in LNs, as well as stage 3/4 precursors able to generate ILC3 or NK cells [27,29,30]. In particular, stage 4 is divided into stage 4a, which is represented by RORγt^+^CD56^+^CD117^+^CD94^−^AhR^+^IL-22 producing ILC3, and stage 4b, characterized by Tbet^+^EOMES^+^CD56^+^CD117^−^CD94^+^ IFN-γ producing NK cells [31]. The mechanisms that rule the ILC differentiation have been extensively studied, and in recent years, several epigenetic mechanisms, such as DNA demethylation and histone acetylation/methylation, have also been suggested to play a role in NK/ILC cell development [32,33,34,35]. Importantly, the characterization of mechanisms modulating NK cell phenotypic and functional maturation may provide new tools for designing NK cell-based immunotherapy protocols, either in consideration of the possibility of improving in vivo NK cell response against tumors, or the possibility of improving the ex vivo generation of NK cells suitable for adoptive cell therapy. In this context, it has been demonstrated that several targeted therapies not only have a direct effect on tumor cells, but also influence the immune system, including NK cell response [36]. Thus, it is important to define whether the targeting of epigenetic DNA modifications may also affect NK cell functional maturation. In the present paper, we analyze the effect of EZH1/2 inhibitors on NK cell in vitro differentiation using a well-established model of human NK cell development that has previously been shown to replicate the steps of NK cell maturation observed ex vivo [37]. Our data suggest that treatment with EZH1/2 sharply induces CD56^+^ cell proliferation, but skews the differentiation lineage commitment towards ILC3.

## 2. Results

### 2.1. EZH1/2 Inhibitors GSK126 and UNC1999 Favor CD56^+^ Cell Proliferation and Skew CD56^+^ Cells Differentiation towards CD56^+^CD117^+^CD94/NKG2A^−^ RORγt^+^ ILC3s

**Figure 1:**
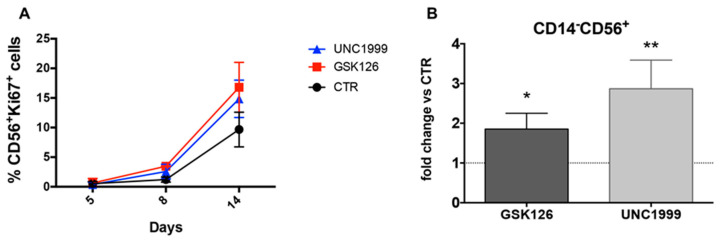
Analyses of CD14^−^CD56^+^ cell precursors proliferation and recovery in cultures performed in the absence or in the presence of EZH1/2 inhibitors. (**A**) Time course analysis shows Ki67 staining in CD14^−^CD56^+^ cells undergoing differentiation from UCB-CD34^+^ in the absence (CTR) or in the presence of GSK126 1 μM (GSK) or UNC1999 1 μM (UNC), between 5 and 14 days of culture. Data obtained from three experiments. (**B**) The histograms represent the fold change of percentages of CD14^−^CD56^+^ cells differentiated in the presence of GSK126 1 μM (GSK) or UNC1999 1 μM (UNC) detectable after 20 days of culture, as compared to CTR condition, arbitrarily normalized to one. Data are expressed as mean values with ± SEM obtained by 10 independent experiments and analyzed by Wilcoxon Signed Rank Test (* *p* < 0.05; ** *p* < 0.005).

**Figure 2:**
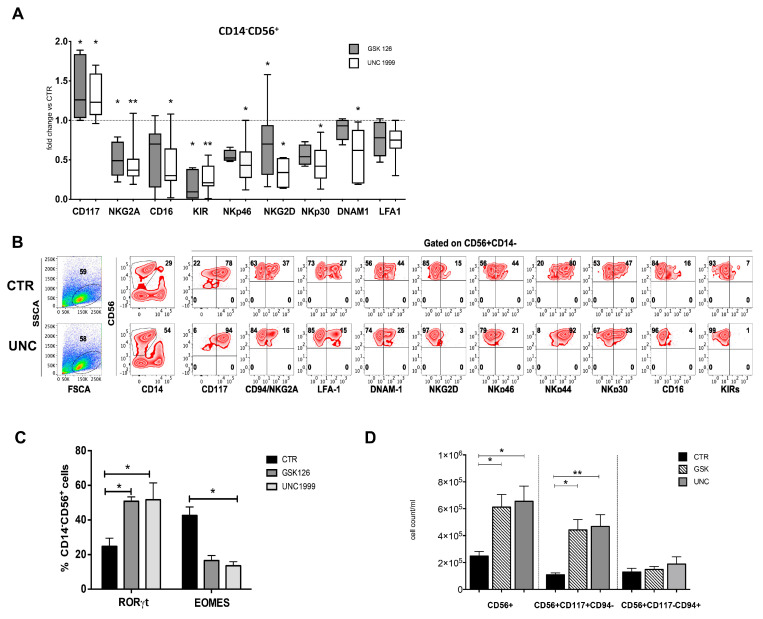
Phenotypic analyses of CD14^−^CD56^+^ cells recovered in the absence or in the presence of UNC1999 and GSK126. (**A**) Box and whisker show the fold change in percentages of CD14^−^CD56^+^ cells expressing CD117, CD94, CD16, KIRs (KIR2DL1, KIR2DL2/DL3, KIR3DL1), NKp46, NKp30, NKG2D, DNAM-1 and LFA-1 in cultures performed in the presence of GSK126 1 μM (GSK) or UNC1999 1 μM (UNC) after 25 days of culture, as compared to CTR condition, arbitrarily normalized to one. Data are obtained by 10 independent experiments and analyzed by Wilcoxon Signed Rank Test (* *p* < 0.05; ** *p* < 0.005). (**B**) Zebra plots show the surface staining of the indicated surface markers, expressed by CD14^−^CD56^+^ cells in the absence (CTR) or in the presence of UNC1999 1 μM (UNC) after 25 days of culture. KIRs indicates the simultaneous staining of anti-KIR2DL1, KIR2DL2/DL3, KIR3DL1 mAbs. Representative experiment out of 10. (**C**) The histograms represent CD14^−^CD56^+^RORγt^+^ and CD14^−^CD56^+^Eomes^+^ cell percentages detected in cultures performed the absence (CTR) or in the presence of GSK126 1 μM (GSK) or UNC1999 1 μM (UNC) after 25 days of culture. Data are expressed as mean values with ± SEM obtained in 9 independent experiments and analyzed by 2wayANOVA Test. (**D**) The histogram shows the cell count/mL of CD56^+^, CD56^+^CD117^+^CD94^−^ and CD56^+^CD117^−^CD94^+^ cells. Cells were harvested after 25 days of culture in the absence (CTR) or in the presence of GSK126 or UNC1999 at 1 μM concentration. The data are represented as the mean values ± SEM obtained by eight independent experiments and analyzed by Kruskal–Wallis test (* *p* < 0.05; ** *p* < 0.005).

To understand whether EZH1/EZH2 enzymatic subunits of PRC1/PRC2 could be involved in the regulation of NK cell differentiation, Umbilical Cord Blood (UCB)-derived CD34^+^ cells were isolated and cultured in an appropriate cytokine mix medium (see Section 4) in the absence (CTR) or in the presence of EZH1/EZH2 inhibitor UNC1999 1 μM (UNC) or selective EZH2 inhibitor GSK126 1 μM (GSK). Time course analyses of the nuclear metabolic marker Ki67, revealed that UNC and GSK favored the proliferation of CD56^+^ cells as compared to CTR after 15 days of culture (Figure 1A). Analyses performed after further 5 days (20 days of culture) confirmed that UNC and GSK significantly favored the recovery of CD14^−^CD56^+^ cells as compared to CTR (Figure 1B). However, the evaluation of NK/ILC-specific surface markers, showed that UNC and GSK significantly enriched in CD56^+^CD117^+^CD94/NKG2A^−^KIR^−^ ILC3-like cells (Figure 2A,B), while the percentages of CD56^+^ expressing CD94/NKG2A, KIR, CD16, NKG2D, NKp30 and NKp46 were significantly reduced in the presence of UNC, as compared to CTR (Figure 2A,B). It is of note that, in control cultures, the CD56^+^LFA-1^+^ cells include both CD56^+^CD94/NKG2A^+^ cells and the terminally differentiated CD56^+^CD16^+^KIR^+^ stage 5 NK cells (Figure S1A). We also performed analysis of Annexin V expression to evaluate whether GSK126 or UNC1999 could induce apoptosis of the different cell subsets detectable in the cultures. As shown in a representative experiments (Figure S1B) the stimulation with both GSK126 and UNC1999 did not increase the percentages of CD56^+^ apoptotic cells and in particular did not affect the viability of CD56^+^CD117^−^CD94/NKG2A^+^ stage IV/V NK cells. 

The expression of Eomes TF or RORγt TF contributes to identify CD56^+^CD117^−^CD94/NKG2A^+^ NK cells of stage 4/5 and CD56^+^CD117^+^CD94/NKG2A^−^ ILC3 cells, respectively. Thus, we compared the expression of Eomes and RORγt TF in CD56^+^ cells undergoing differentiation in the absence or in the presence of EZH1/2 inhibitors. Our results show that both EZH1/2 inhibitors led to increase percentages of CD56^+^RORγt^+^ cells, while significantly reduced the percentages of CD56^+^Eomes^+^ cells as compared to CTR (Figure 2C). The cell counts performed after 25 days of culture indicated that the presence of EZH1/2 inhibitors did not reduce the CD56^+^CD117^−^CD94/NKG2A^+^ cell numbers, but rather significantly increased the numbers of CD56^+^CD117^+^CD94/NKG2A^−^ as compared to control cultures (Figure 2D and Figure S1C).

### 2.2. EZH1/2 Inhibitors Do Not Affect Cytokines Expression but Reduce Frequencies of CD56^+^ Cells with Cytolytic Potential

**Figure 3:**
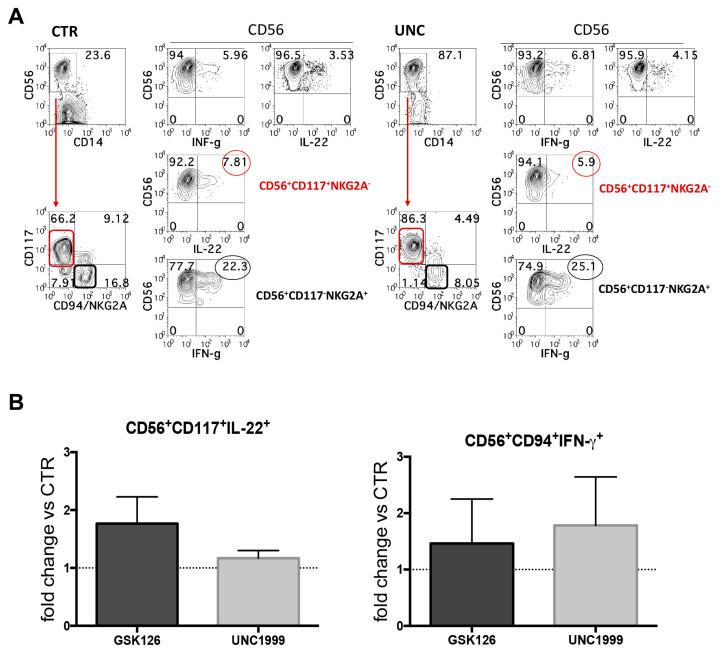
Cytofluorimetric analyses of intracytoplasmic cytokines in CD14^−^CD56^+^ cells that underwent differentiation in the absence or in the presence of UNC1999 or GSK126. (**A**,**B**) After 25 days of culture, cells were stimulated overnight with IL-1β+IL-7+IL23 or IL12+IL-15+IL-18 to induce the expression of IL-22 or IFN-γ respectively. (**A**) Upper contour plots show the intracytoplasmic staining of IL-22 and IFN-γ cytokines expressed by CD14^−^CD56^+^ cells. Lower contour plots show the percentages of CD14^−^CD56^+^CD117^+^CD94/NKG2A-IL-22^+^cells (red) and CD14^−^CD56^+^CD117^−^CD94/NKG2A^+^IFN-γ^+^ cells (black) detectable in cultures performed in the absence (CTR) or in the presence of UNC1999 1 μM (UNC). Representative experiment out of five. (**B**) The histograms show the fold change of CD14^−^CD56^+^CD117^+^CD94/NKG2A^−^IL-22^+^ and CD14^−^CD56^+^CD117^−^CD94/NKG2A^+^IFN-γ^+^ cells detected in cultures performed in the presence of GSK126 1 μM (GSK) or UNC1999 1 μM (UNC) after 25 days of culture as compared to CTR, arbitrarily normalized to one. Data are expressed as mean values ± SEM obtained in five independent experiments and analyzed by Wilcoxon Signed Rank Test.

**Figure 4:**
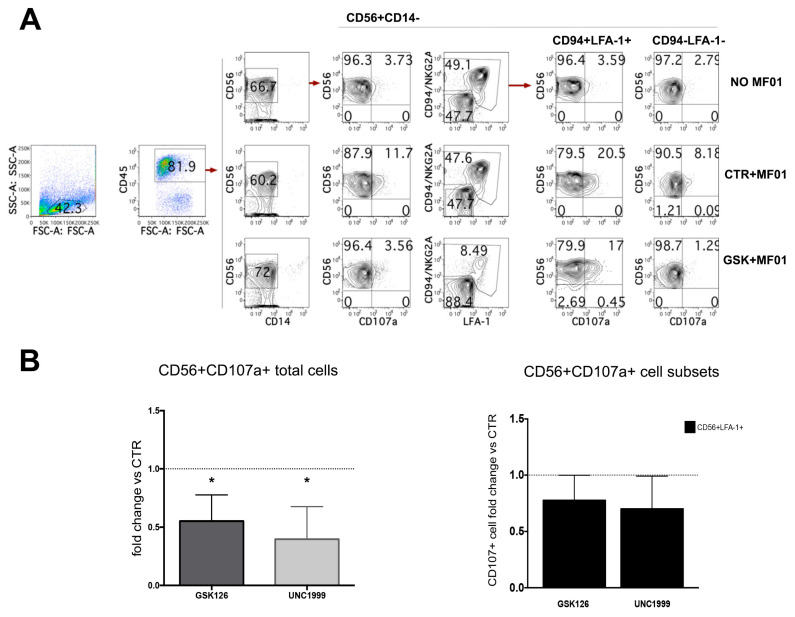
After 27 days of culture, CD14^−^CD56^+^ cells were incubated with the NK-susceptible human melanoma cell line MFO1 and analyzed for the expression of CD107a. (**A**) Dot Plots show CD107a staining on CD45^+^CD56^+^CD14^−^ cells and on relative LFA-1^+^CD94^+^ and CD94^−^LFA-1^−^ cell subsets after incubation with MFO1 cell lines. A representative experiment performed with GSK126 is shown. (**B**) The left histogram shows the fold change of total CD56^+^CD107a^+^ cell percentages detectable in cultures performed the presence of GSK126 1 μM (GSK) or UNC1999 1 μM (UNC) as compared to CTR condition, arbitrarily normalized to one. The right histogram represents the fold change of CD56^+^CD94^+^LFA-1^+^ CD107a^+^ cell percentages detectable in cultures performed the presence of GSK126 1 μM (GSK) or UNC1999 1 μM (UNC) as compared to CTR condition, arbitrarily normalized to one. Effector/target (E/T) cell ratio was 1/1 (see Methods section). Data are expressed as mean values ± SD obtained by eight independent experiments and analyzed by Wilcoxon Signed Rank Test (* *p* < 0.05).

NK cells can both produce IFN-γ and exert cytolytic activity against tumor cells, while ILC3s are regulatory cells, which produce IL-22. Since after 25 days of culture we obtained two main populations, namely CD56^+^CD117^+^CD94/NKG2A^−^RORγt^+^ILC3 and CD56^+^CD117^−^CD94/NKG2A^+^Eomes^+^NK cells, we analyzed their ability to express IL-22 and IFN-γ intra-cytoplasmic cytokines upon stimulation with appropriate cytokines, i.e., IL-1β+IL-7+IL-23 or IL-12+IL-15+IL-18, respectively. The results suggested that there was a great variability among different experiments but no significant differences could be detected when compared to CTR (Figure 3A,B). We also evaluated the cytolytic potential of CD56^+^ cells generated in the absence or in the presence of GSK126 or UNC1999 by performing the CD107a degranulation assay. Our analyses revealed that CD56^+^ cells obtained from GSK- and UNC-conditioned cultures expressed lower percentages of CD107a^+^ cells upon incubation with the NK-susceptible human melanoma cell line MFO1, as compared to CTR (percentage range CD56^+^CD107a^+^ cells: CTR = 8–21; GSK126 = 1.6–13; UNC1999 = 1–13.1) (Figure 4A,B). Of note, when we analyzed the expression of CD107a specifically on the potential cytotoxic cell subset, namely CD56^+^LFA-1^+^CD94^+^ NK cells, we observed a decrease of CD107a^+^ NK cells in cultures performed in the presence of EZH1/2 inhibitors as compared to control cultures; however, there was a great variability among experiments and results were not significant (Figure 4A,B). Similar data were also obtained with other NK-susceptible human tumor cells such as K562 human erythroleukemia cell line (Figure S2A). We analyzed whether treatment with EZH1/2 inhibitors could dampen the expression of Perforin in CD56^+^LFA-1^+^CD94^+^ NK cells, and overall, the treatment with EZH1/2 led to the generation of NK cells displaying a decrease of CD56^+^Perforin^+^ cell percentages as compared to controls that however was not significant (Figure S2B,C). 

### 2.3. GSK126 and UNC1999 Skewed CD34^+^ HSPC Commitment towards CD117^+^CD127^+^AhR^+^ILC3 Precursors

**Figure 5:**
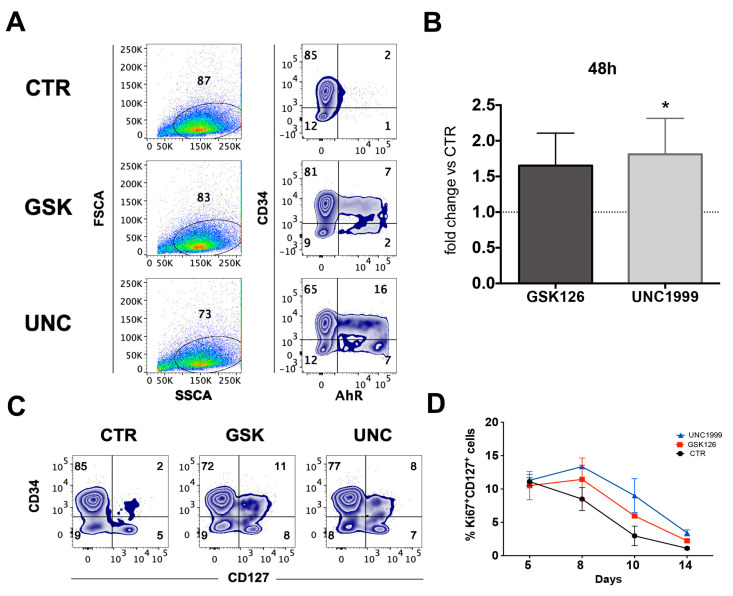
Early time intervals analyses of AhR TF and CD107a surface marker in UCB-CD34^+^ cell precursors cultured in the absence or in the presence of UNC1999 and GSK126. (**A**) Zebra plots show the intranuclear staining of AhR TF expressed by UCB-CD34^+^ cell precursors in the absence (CTR) or in the presence of GSK126 1 μM (GSK) or UNC1999 1 μM (UNC) after 48 h of culture. Representative experiment out of six. (**B**) The histograms represent the fold change of CD34^+^CD117^+^AhR^+^ cell percentages detectable in the presence of GSK126 1 μM (GSK) or UNC1999 1μM (UNC) after 48 h of culture as compared to CTR condition, arbitrarily normalized to one. Data are expressed as mean values ± SEM obtained by of 6 independent experiments. (**C**) Zebra plots show the surface staining of CD127 (IL-7Rα) expressed by CD34^+^ cells in the absence (CTR) or in the presence of GSK126 1 μM (GSK) or UNC1999 1 μM (UNC) after 48 h of culture. Representative experiment out of four. (**D**) Time course shows the percentages of CD127^+^ cells stained with Ki67 proliferation marker in the absence or in the presence of GSK126 1 μM (GSK) or UNC1999 1 μM (UNC) between 5 and 14 days of culture. Data are the mean values ± SEM of two independent experiments.

**Figure 6:**
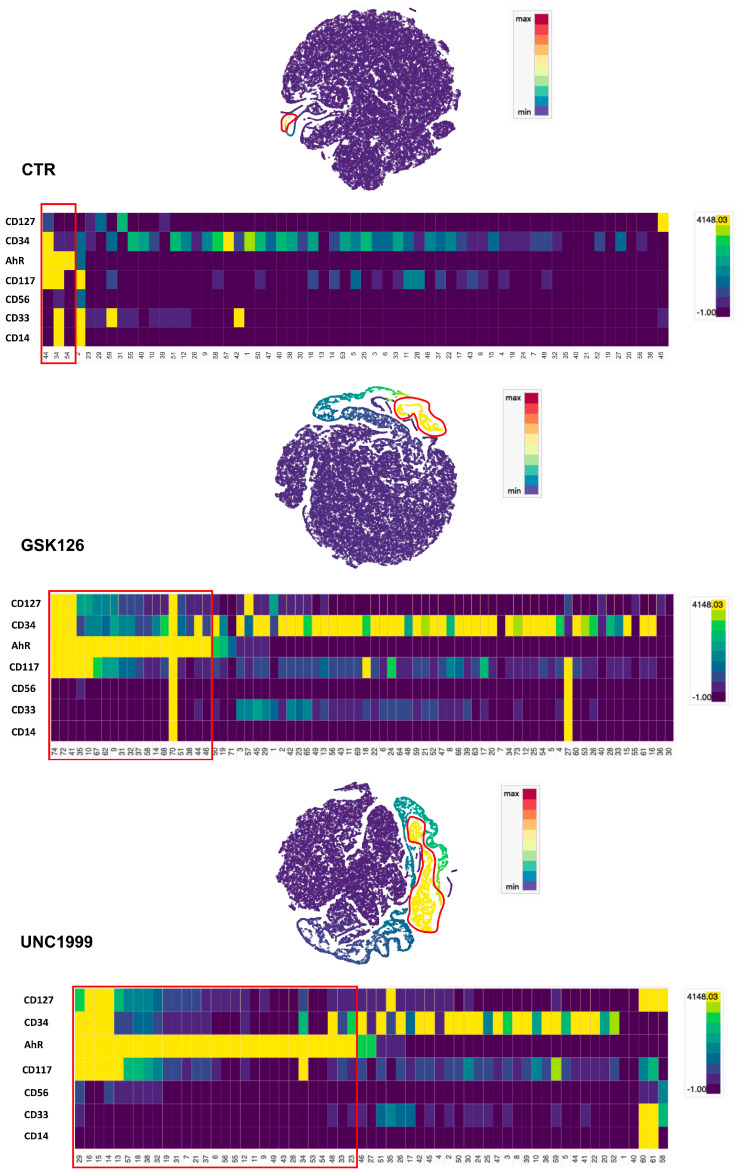
The t-SNE embedding analyses of purified UCB-CD34^+^ cell precursors after 48 h of culture. The t-SNEs show the AhR TF expression by single cells from Min (Blue) to Max (Red). The heatmaps show the intensity expression of indicative surface markers (CD127, CD34, AhR, CD117, CD56, CD33 and CD14) in each cluster identified (representative experiment). The evaluation of indicative marker intensity expression in each cluster is shown by the heatmaps. In the t-SNE plots and Heatmaps, clusters with the highest expression of AhR are highlighted in red. The t-SNE analysis was generated by Cytosplore software.

**Figure 7:**
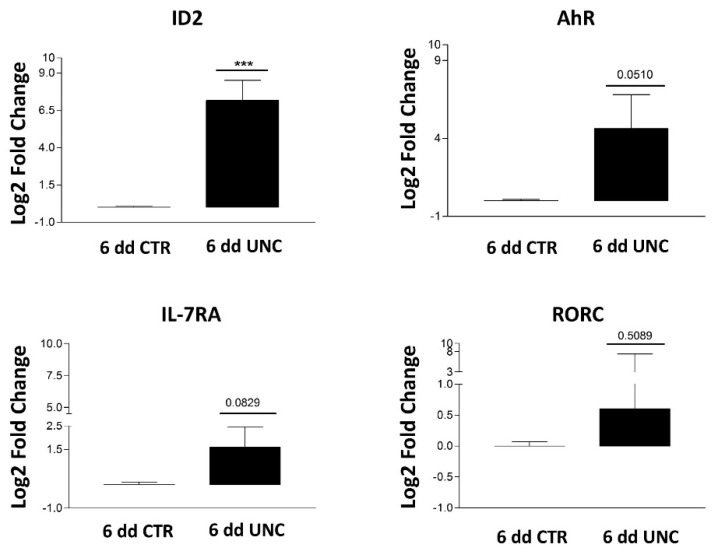
RT-qPCR analyses of the effects of UNC treatment on purified UCB-CD34^+^ cell precursors undergoing differentiation after 6 days of culture in the absence or in the presence of UNC 1999. Results are presented as Log2 fold change versus CD34^+^ cell control T0′ and 6 days (dd), respectively of relative quantification analysis efficiency method based on gene target compared to the 18S rRNA and ACTB housekeeping genes. Values are the mean ± SEM of RT-qPCR analyses on three different biological replicates. (*** *p* < 0.001).

Our results suggest that the enrichment in ILC3 cells could be due to a UNC- or GSK-mediated effect on HSPC commitment towards ILC3 rather than NK cells. Thus, we plated UCB-CD34^+^ in the absence (CTR) or in the presence of GSK126 or UNC1999 and we performed cytofluorimetric analyses at early time culture intervals (48–72 and 120 h) to detect the expression of markers that have been shown to correlate with ILC3 commitment, such as aryl hydrocarbon receptor (AhR) TF and CD127 (IL-7Rα) [38,39]. The results showed that, in the presence of GSK and UNC, it was possible to detect CD117^+^AhR^+^ precursors that were undetectable in CTR already after 48 h of culture (Figure 5A,B). Since CD127 (IL-7Rα) surface expression was poorly detectable, we also cultured CD34^+^ cells with medium enriched only in SCF and FLT3-L cytokines in the absence of IL-7 (to better detect the expression of CD127 on the cell membrane). The results revealed an increase in CD34^+^ cell percentages expressing CD127 upon conditioning with GSK or UNC as compared to CTR after 48 h of culture (Figure 5C). Accordingly, time course analysis of CD127 expression on CD56^+^Ki67^+^ proliferating cells suggested that UNC and GSK would favor the metabolic activation of CD56^+^CD127^+^ cells as compared to CTR in the first two weeks of culture (Figure 5D). We performed t-distributed stochastic neighbor (t-SNE) embedding of collective cells analyses performed of UCB-CD34^+^ cell precursors after 48 h of culture, and we detected the presence of multiple clusters expressing AhR TF in GSK- and UNC-conditioned cultures that were not detectable in CTR culture. Of note, clusters expressing CD117, CD127 and AhR were observed only in GSK and UNC conditions (Figure 6). To confirm these data, we also performed RT-qPCR analyses to detect ID2, AhR, RORC or CD127 mRNA expression in UCB-CD34^+^ cell precursors cultured for six days with appropriate cytokines in the in the absence (CTR) or in the presence of UNC1999. Our results suggest that the presence of UNC can lead to an increased expression of ILC/NK ID2 common TF mRNA but also of ILC3-specific AhR TF mRNA and to a slight increase of RORC mRNA and CD127 mRNA as compared to control cultures (Figure 7).

### 2.4. Repeated UNC1999 Addition to Cell Cultures Is Required to Skew CD56^+^ Cell Differentiation towards ILC3

**Figure 8:**
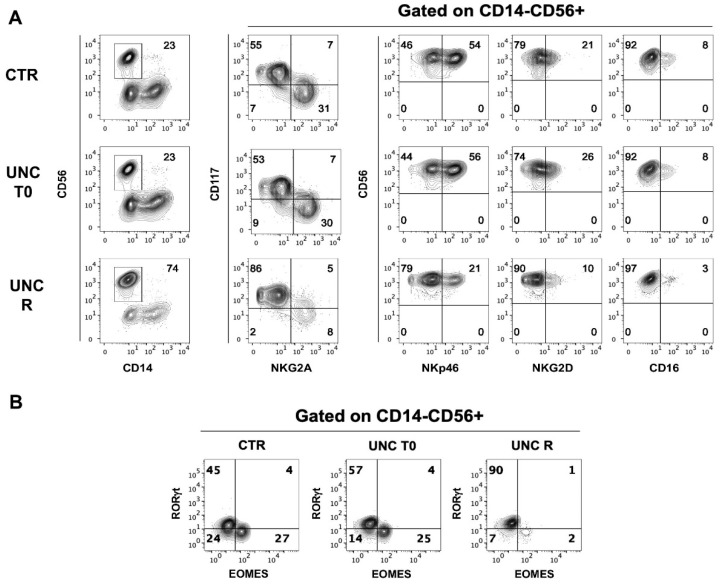
Phenotypic analyses of CD56^+^ cells generated in the absence or in the presence of UNC1999 added at different time intervals. (**A**) Flow cytometric analyses show surface staining of surface markers expressed by CD14^−^CD56^+^ cells obtained after 25 days of culture in the absence (CTR) or in the presence of UNC1999 1 μM (UNC) added at the beginning of the culture (UNC T0) or added twice/week to the cell culture (UNC R). Representative experiments out of five. (**B**) Contour plots show the intranuclear staining of Eomes and RORγt TF expressed by CD14^−^CD56^+^ cells obtained after 25 days of culture in the absence (CTR) or in the presence of UNC1999 1 μM (UNC) added at the beginning of the culture (UNC T0) or added as a chronic simulation (UNC R). Representative experiments out of five.

Yin et al. demonstrated that Ezh2-deficient mice show an increase of the generation of mature NK cells expressing higher levels of NKG2D activating receptor, Granzyme B, CD122 and CD127. These authors also performed experiments on human UCB-CD34^+^ precursors undergoing in vitro NK cell development in the presence of a single stimulation with UNC1999 at the beginning of the culture. Their results appeared to be in agreement with the results obtained with the murine model [40]. Given the striking differences from our data, we verified the effect of the addition of UNC1999 only at the beginning of the culture to allow a comparison with our data obtained in the presence of repeated addition (twice/week) of EZH1/2 to the cultures. We cultured UCB-CD34^+^ precursors for 25 days by stimulating them with a repeated or a single (i.e., at the beginning of the culture) exposure to EZH1/2 UNC inhibitor. As shown by a representative experiment (Figure 8A) only the repeated stimulation led to the enrichment in CD56^+^CD117^+^ ILC3 after 25 days of culture, while in the condition of a single stimulation, the repertoire of NK/ILC innate lymphoid cells was similar to that observed in the CTR culture. Analyses of TF confirmed that only the repeated stimulation with UNC increased the percentages of CD56^+^RORγt^+^ cells, while Eomes remained poorly expressed (Figure 8B).

## 3. Discussion

In the present study, we analyzed the effects of EZH1/2 inhibitor treatment on in vitro NK cell development. We show that the continued exposure to these compounds shift the commitment of CD34^+^ HSPC towards CD56^+^CD117^+^LFA-1^−^CD94/NKG2A^−^RORγt^+^ ILC3, reducing the cell frequency of CD56^+^CD117^−^LFA-1^+^CD94/NKG2A^+^ fully cytotoxic NK cells.

Natural killer lymphocytes may represent important effector cells in cancer immunotherapy, particularly in the control of hematological malignancies [41]. There is also evidence that cytotoxic NK cells may positively correlate with better prognosis of different solid tumors such as melanoma, but their exploitation in such diseases is still limited [21,42,43,44,45,46,47]. However, the combination of NK cell-based immunotherapies with other innovative anticancer therapies might provide a synergistic effect against tumor growth and metastasis [48,49]. In this context, DNA epigenetic modifications have been shown to play a primary role in the progression of both hematological and solid tumors. EZH1 and EZH2 methyltransferases, regulating H3K27me3, represent one of these targets suitable for developing novel therapeutic strategies for cancer therapy: they are involved both in normal and malignant hematopoiesis [3,50], and EZH2 dysregulation also occurs frequently in solid tumors such as melanoma, prostate, ovarian and lung cancers [5,51,52]. However, the effects of such epigenetic modulation on immune cells, and in particular on NK cell maturation, should be carefully evaluated to design effective combined therapeutic protocols [53].

To this end, we analyzed the effect of EZH2-specific inhibitor GSK126 and EZH1/2-specific inhibitor UNC1999 on NK cell maturation in a well-established model of in vitro NK/ILC cell development and functional maturation. Ki67 analysis and cell counts revealed that the inhibition of EZH1/2 leads to a significant increase in recovery of CD56^+^ cells. However, phenotypic analyses showed sharp differences from control cultures. In particular, both the exposure to GSK126 and UNC1999 induced a reduction of percentages of CD56^+^CD117^−^CD94/NKG2A^+^NCR^+^stage 4 NK cells, and in particular of CD56^+^CD16^+^KIR^+^ stage 5 terminally differentiated NK cells, while there was an increase of CD56^+^CD117^+^LFA-1^−^CD94/NKG2A^−^ stage 3 immature NK cell percentages as compared to controls. Analyses of TF expression indicated that EZH1/2 inhibition also leads to a sharp reduction of CD56^+^Eomes^+^ NK cell frequency, favoring the enrichment of CD56^+^RORγt^+^ cell percentages, suggesting a prevalence of ILC3 among the CD56^+^CD117^+^LFA-1^−^CD94/NKG2A^−^ cells. Of note, cell counts performed after 25 days of culture indicated that, despite the significant higher CD56^+^ cell recovery observed in the presence of EZH1/2 inhibitors, only those of the CD56^+^CD117^+^CD94/NKG2A^−^ cell subset displayed a significant increase in cell recovery, while the numbers of CD56^+^CD117^−^CD94/NKG2A^+^ did not significantly change as compared to controls. It is conceivable that these inhibitors do not impaired the development of stage 4 NK cells (i.e., CD14^−^CD56^+^LFA-1^+^ CD94/NKG2A^+^), but may impair the acquisition of KIR receptors (stage 5 NK cells), as CD56^+^KIR^+^ cells were virtually undetectable in cultures performed with EZH1/2 inhibitors.

The functional analyses of CD56^+^ cell subsets, generated in the absence or in the presence of EZH1/2 inhibitors, showed that the treatment with both inhibitors did not significantly change the expression of IL-22 or IFN-γ by the specific subsets CD56^+^CD117^+^LFA-1^−^CD94/NKG2A^−^ cells and CD56^+^CD117^−^LFA-1^+^CD94/NKG2A^+^ cell subsets, respectively, as compared to controls. Thus, in our model, the inhibition of EZH1/2 does not influence the acquisition of the ability to produce cytokines. It should be considered that the presence of cells able to produce these cytokines may play a dual role in tumor control, possibly eliciting negative feedback mechanisms, such as the increased transcription of the immunosuppressive enzyme Indoleamine-pyrrole 2,3-dioxygenase (IDO) by tumor cells in the presence of IFN-γ release [54,55]. Moreover, the role of the ILC3 producing IL-22 in promoting rather than controlling tumor progression is also still uncertain [56,57,58].

On the other hand, analyses of cytotoxic potential evaluated by CD107a degranulation assay showed that CD56^+^ cells generated in the presence of GSK126 or UNC1999 display a significant reduction of CD107a^+^ cell percentages upon engagement with the NK-susceptible MFO1 human melanoma cell line as compared to controls. Detailed analysis of the mature CD56^+^CD94^+^LFA-1^+^ potentially cytotoxic NK cells indicated that cells developed in the presence of EZH1/2 inhibitors may display a slight decrease of CD107a^+^ cells upon incubation with tumor cell lines as compared to controls. Accordingly, data on intracytoplasmic perforin expression would suggest the possibility that EZH1/2 inhibitors could interfere with the generation of fully cytotoxic NK cells. It is possible that the reduction of cytolytic potential of CD56^+^ cells could be due to both the shift in proliferation towards ILC3, which reduced frequencies of the most mature CD56^+^LFA-1^+^ CD94/NKG2A^+^CD16^+^KIR^+^ NK cells among CD56^+^ cells, and to partial reduction of Perforin content.

Taken together, these data indicate that UNC1999 and GSK126 pushed CD56^+^ cell expansion from UCB-CD34^+^ cell precursors; however, they did not increase the generation of mature NK cells but rather of CD56^+^ILC3, leading to the generation of non-cytolytic ILCs. This should be taken into account in view of designing novel protocols to improve ex vivo generation of cytotoxic NK cells for adoptive NK cell therapy and should also be carefully evaluated when the use of these drugs is considered not only for halting tumor growth and progression, but also for improving anti-tumor immune responses. It has been reported in in vitro experiments that EZH2 inhibition increases the expression of NKG2D ligands on hepatocarcinoma cell lines that then became more susceptible to NK cell-mediated lysis [59]. However, it is conceivable that, in in vivo treatment, both tumor cells and NK cells and their precursors will be exposed to the same drug, both becoming susceptible to the effect of EZH2 inhibitor, and thus leading to different results. In this context, we observed reduced frequencies in the cultures of cytotoxic CD56^+^NKG2D^+^ NK cells upon in vitro treatment with both EZH1/2 inhibitors. It is of note that in our experiments, both inhibitors displayed overlapping effects; however, the most significant results were obtained in the presence of the EZH1/2 inhibitor UNC1999, suggesting that the impairment of both EZH1 and EZH2 subunits leads to a most sharp effect.

It appeared conceivable that the increase of ILC3 cells observed in the presence of both inhibitors could be due to a key role exerted by EZH1/2 on ILC/NK cell lineage commitment. Our analyses of UCB-CD34^+^ cell precursors cultured for 48 h in the presence of GSK126 or UNC1999 showed an increase of AhR^+^ and of CD127^+^ cell precursors as compared to control. In particular, tSNE analyses suggested that, in the presence of both inhibitors, it was possible to detect several clusters expressing AhR, as compared to controls. Of note, some of them also displayed the simultaneous expression of CD127 and a close reciprocal proximity in tSNE. Accordingly, molecular analyses also confirmed an increased expression of ID2, AhR and RORC mRNA expression after 6 days of culture in the presence of UNC1999. Both AhR TF and CD127 (IL7-Rα) are well known to be involved in ILC3 cell development [38,39]: in particular, several papers have reported the key role of AhR not only in favoring the development of ILC3 but also in the inhibition of NK cell terminal differentiation [60,61]. It has been shown that AML blasts may induce AhR expression in NK cell precursors, impairing their maturation and functions, and thus favoring tumor escape from NK cell-mediated control [61].

Yin et al. reported that Ezh2-deficient mice show an increased generation of mature NK cells expressing higher levels of NKG2D activating receptor, Granzyme B, CD122 and CD127. They also reported some in vitro data on human UCB-CD34^+^ cell precursors undergoing in vitro NK cell differentiation in the absence or in the presence of UNC1999 [40]. These results, as in the murine model, would suggest that the impairment of EZH2 would increase the percentages of cytotoxic CD56^+^NKp46^+^NKG2D^+^ NK cells [40]. These data are clearly in contrast with our results. Of note, their model of NK differentiation seems to differ from ours because they added UNC1999 only once, at the beginning of the culture. For this reason, we replicated their experiments to compare the results with those obtained using our model, which requires a repeated administration of UNC1999. The results clearly indicated that a single stimulation at the beginning of the culture did not give any advantage to NK cell generation compared to controls. Moreover, the sharp skewing of precursor commitment towards ILC3 was possible only upon continuous inhibition of EZH1/2.

We could speculate that patients undergoing to this kind of therapies may be exposed to repeated drug administration, thus paralleling our in vitro system and possibly skewing ILC development towards ILC3. It is important also to remind that NK cells may give an important contribution to solid tumor immunosurveillance also during metastatic spread through LNs, where it is possible to detect NK cell precursors and where the majority of mature NK cells are represented by the non-cytotoxic CD56^bright^CD16^−^ cells subset [24,30,62]. These NK cells would need appropriate stimuli to complete their maturation and to acquire anti-tumor cell cytotoxicity [63,64]. Thus, in designing combined therapeutic treatments, it would be important to identify combinations that could improve cell cytotoxicity. On the other hand, at least for GSK126, a short plasma half-life has been reported, suggesting that the compound could have a limited effect on NK cell maturation [65].

It has been reported that in some hematological malignancies, such as AML, it is possible to detect a defective NK cell mature repertoire [66]. This could be due to tumor microenvironment conditions such as alteration of hematopoietic bone marrow niche or soluble factors released by the tumors that would interfere with NK cell maturation [67,68,69]. However, it would be of interest to verify whether epigenetic dysregulation may play a role in this phenomenon, particularly if the possibility of treating these patients with epigenetic modulators would be taken into consideration. Unfortunately, in our model, it is only possible to assess with a good reliability the effects of epigenetic modulation at the early stages of ILC/NK cell commitment, mainly because in the presence of EZH1/2 inhibitors we do not observe the expansion of significant amounts of CD56^+^CD94^+^CD16^+^KIR^+^ NK cells. However, it would also be of interest to evaluate potential effects on NK cells terminally differentiated (CD57^+^) and to monitor ex vivo the repertoire of NK cells in patients undergoing these therapeutic treatments.

## 4. Materials and Methods

### 4.1. Cell Isolation and In Vitro Culture

Liguria Cord Blood Bank selected and provided Umbilical Cord Blood (UCB) samples from healthy individuals. Liguria Regional Ethical Committee approved the study and mothers gave their written informed consent according to the Helsinki Declaration. Mononuclear cells were obtained by Ficoll-Lympholyte separation (Cedarlane, Burlington, Canada). CD56^−^CD34^+^ cells (>98% purity) were obtained by MACS positive separation (Miltenyi, Bergisch Gladbach, Germany) and controlled by FACS. Cells were cultured in RPMI 1640 (Lonza, Basel, Belgium) containing 10% human AB serum (Biowest, Nuaillè, France), 1% glutamine and 1% of penicillin, neomycin and streptomycin antibiotic mixture, Stem Cell Factor (SCF) (10 ng/mL), Fms-related tyrosine kinase 3 ligand (Flt3-L) (10 ng/mL), Interleukin-7 (IL-7) (20 ng/mL), Interleukin-15 (IL-15) (20 ng/mL), Interleukin-21 (IL-21) (20 ng/mL) (Miltenyi, Bergisch Gladbach, Germany), in the absence or in the presence of UNC1999 (UNC) or GSK126 (GSK) at 1 μM concentration (Selleck Chemicals, Houston, TX, USA). We added UNC1999 and GSK126 at day 0 and after 1 week, twice a week. After 25 days of culture cells obtained in the different culture conditions were harvested and counted to further perform functional analyses. The drug concentrations were chosen after having performed preliminary UCB-CD34^+^ cell cultures performed with escalating doses of UNC1999 (3-1 μM) and analyzed surface phenotype and cell recovery after 15 days of culture (Figure S3A surface phenotype, Figure S3B absolute cell counts). Since manufacturer’s indications suggested similar doses of GSK126 to be used in in vitro experiments, we decided to use the same drug concentration for both inhibitors.

### 4.2. Monoclonal Antibodies (mAbs) and Flow Cytometry

All the mAbs used were mouse-anti human. The mAb CD56 (Clone: N901) PeCy7 conjugated, CD159a (clone: Z199) APC conjugated, CD158a (Clone: EB6B) APC conjugated, CD158b1/b2,j (Clone: GL183) APC conjugated and CD158e1/e2 (Clone: Z27.3.7) APC conjugated were purchased from Beckman-Coulter (Brea, CA, USA). The mAb CD33 (clone: REA775) VioGreen conjugated, CD14 (Clone: TÜK4) FITC conjugated, CD16 (Clone: REA423) FITC conjugated and CD107a (Clone: LAMP-1) PE conjugated were purchased from Miltenyi Biotec, Bergisch Gladbach, Germany). The mAb CD14 (Clone: 61D3) APC-eFluor780 conjugated, CD117 (Clone: 104D29) Brilliant Violet 421 conjugated, CD34 (Clone: 4H11) PE conjugated, AhR (Clone: FF3399) PE conjugated, RORγt (Clone: AFKJS-9) PE conjugated, IL-22 (Clone: 22URTI) PE conjugated, Eomes (Clone: WD1928) eFluor-660 conjugated, IFN-γ (Clone: 4S.B3) eFluor 450 conjugated and Perforin (Clone: dG9) PE conjugated were purchased from eBioscience–ThermoFisher (Waltham, MA, USA). The mAb CD16 (Clone: 3G8) Brilliant Violet 421 conjugated, CD127 (Clone: A019D5) Brilliant Violet 421 conjugated, CD226 DNAM-1 (Clone: 11A8) PE conjugated, CD335 NKp46 (Clone: 9E2) eFluor 450 conjugated, CD336 NKp44 (Clone: P44-8) Alexa Fluor 647 conjugated and CD337 NKp30 (Clone: P30-15) Alexa Fluor 647 conjugated were purchased from BioLegend (San Diego, CA, USA). To assess cell proliferation, we performed analyses with mAb Ki67 (Clone: B56) PerCP-Cy 5.5 conjugated (BD Biosciences, San Jose, CA, USA). To assess cell viability, we performed analyses with Propidium Iodide and Annexin V staining (Immunostep, Salamanca, Spain) on cells cultured in the absence or in the presence of EZH1/2 inhibitors. Flow cytometric analyses were performed on MACSQuant analyzer Miltenyi.

### 4.3. t-Distibuted Stochastic Neighbor (t-SNE) Analysis

The t-SNE analyses were performed thanks to Cytosplore software (Cytosplore project; Computer Graphics and Visualization Group at the TU Delft and the Computational Biology Center, the Division of Image Processing, and the Immunohematology and Blood Transfusion Department at the Leiden University Medical Center, Netherlands Delft; Netherlands) [70]. The purified UCB-CD34^+^ cells were cultured in the absence or in the presence of UNC or GSK inhibitors at 1 μM. After 48 h of culture, collective cells (4 × 10^4^ cells/culture condition) were marked with CD34, CD33, CD117, CD127, CD56, CD14 and AhR mAb and analyzed by flow cytometer. The FCS files were analyzed with Cytosplore software. The t-SNE analysis was realized using as k-nearest neighbor (k-NN) metric the Euclidean algorithm and perplexity 15.

### 4.4. Intra-Cytoplasmic Cytokine, Cytolitic Granules and TF Expression Assays

To detect cytokines, cells were stimulated overnight with IL-12 (10 ng/mL), IL-15 (50 ng/mL), IL-18 (100 ng/mL) or IL-1β (50 ng/mL), IL-7 (50 ng/mL), IL-23 (50 ng/mL) (Miltenyi Biotec, Bergisch Gladbach, Germany) in the presence of monensin (GolgiStop) or brefeldin (GolgiPlug) (BD Biosciences), respectively.

For intra-cytoplasmic cytokine and cytolytic granules analyses, cells were stained for surface markers and then fixed and permeabilized with Fixation and Permeabilization Kit (BD Biosciences). Then, cells were incubated with cytokine- or Perforin-specific mAbs. To detect TF expression, cells were suspended in 5% BSA buffer, stained for surface markers, subsequently fixed with Transcription Factor Staining Buffer Set and stained for Eomes, AhR and RORγt mAbs.

CD107a degranulation assay-CD56^+^ obtained lymphocytes in vitro after 27 days of culture were incubated in 1/1 ratio with the melanoma cell line MFO1 or human leukemic cell line K562 in the presence of CD107a mAb for 3-h. Monensin (GolgiStop) was added after one hour of incubation to the cells. At the end of the incubation, the cells were collected and marked to surface immunofluorescence and analyzed on the flow cytometer. In each experiment we performed a comparison between CTR and UNC- or GSK-treated cells and then normalized data to compare all experiment together.

### 4.5. RT-qPCR Analyses

Total RNA was extracted from purified UCB-CD34^+^ cells at T0′ and after 48 h of culture in the absence (CTR) or in the presence of GSK126 or UNC1999 at 1 μM concentration using RNeasy plus mini kit (Qiagen, Hilden, Germany). Relative expression analysis was performed by RT-qPCR into Eppendorf realplex⁴ mastercycler using Sso Advanced Universal SYBR Green Supermix (Biorad, Hercules, CA, USA). cDNA was synthesized from 100 ng of total RNA with iScript cDNA Synthesis kit (Biorad). For conventional RT-qPCR analysis, two microliters of cDNA were amplified by using the following primers for ID2, RORC, AHR and IL7RA analysis. Sequences of ID2 primers were: ACCCTCAACACGGATATCAGC, Forward, CCACACAGTGCTTTGCTGTC, Reverse; RORC primers were: TCTCTGCAAGACTCATCGCC, Forward, TCCACATGCTGGCTACACAG, Reverse. AHR primers were: CAGTACTGCCAGGCCAACAG, Forward, TGGCTGAAGATGTGTGGTAGTC, Reverse. IL7RA primers were: GCCTATCGTATGGCCCAGTC, Forward, GCAGTCCAGGAAACTTTCAGG, Reverse. Relative quantification of mRNAs was calculated by the Ct method. Sequences of internal control primers were: TAGAGGGACAAGTGGCGTTC Forward, CGCTGAGCCAGTCAGTGTAG Reverse, for 18s rRNA, TGCCCTGAGGCACTCTTC Forward, TGAAGGTAGTTTCGTGGATGC Reverse for ACTB. Standardization of target genes was obtained by using β-actin and, 18s rRNA genes as internal control. Values are presented as the mean ± SEM of n = 3 independent RT-qPCR analyses.

Primers were designed using Primer3 software (Howard Hughes Medical Institute and by the National Institutes of Health, National Human Genome Research Institute. Ongoing development is partly funded by the Estonian Ministry of Education and Research and by Centre of Excellence in Genomics and Translational Medicine at University of Tartu, Estonian; http://bioinfo.ut.ee/primer3/) and checked for secondary structures of the amplicon using mfold (http://mfold.rna.albany.edu/?q=mfold).

qPCR was performed on realplex⁴ mastercycler (Eppendorf, Hamburg, Germany) using 5 µL of iTaq universal SYBR Green supermix(2x) (Biorad), 2 µL of cDNA, 0.5 µmol sense and antisense primers in a final reaction volume of 10 µL. After amplification, melting curves with 65 steps of 30 s and 0.5 °C increase were performed. Expression data were normalized on the bestkeeper of 18s rRNA and ACTB gene expression data. Bestkeeper software (FML-Weihenstephan, Centre of Life and Food Science, Technical University of Munich, Germany) was used to determine the stable housekeeping genes relative expression values with standard errors and statistical comparisons (one way analysis of variance) were obtained using Qgene software Qgene software (Department of Plant Breeding and Biometry, Cornell University, Ithaca, NY, USA).

### 4.6. Statistical Analysis

The statistical analysis was performed using Prism GraphPad software (GraphPad Company, San Diego, CA). *p* < 0.05 were considered significant. We used Wilcoxon signed rank test, the Two-way Anova multiple comparison test (Tukey multiple comparison tests), and the One-way Anova Kruskal–Wallis multiple comparison test.

## 5. Conclusions

The development of novel and promising therapeutic approaches in solid tumors, such as epigenetic DNA targeting, has prompted scientists to extensively evaluate the effects of these approaches on the restoring/impairing of T cell-mediated anti-tumor responses [15,16,71,72], while there are still limited data on their effect on NK cell anti-tumor responses. However, the recent progress in the characterization of NK cell biology has made it possible to also establish novel NK-cell-based immunotherapies in combination with these therapies, suggesting the necessity to analyze their effects also on NK cell biology. Our data show that EZH1/2 activity is clearly involved in ILC/NK cell lineage commitment from CD34^+^ precursors, and suggest that, in patients undergoing these therapeutic protocols, ex vivo monitoring of NK cell repertoire and the schedule of EZH1/2 inhibitors administration should be carefully evaluated, because there some side effects, such as the dampening of the generation of fully competent cytotoxic NK cells, could be detected.

## Data Availability

The data presented in this study are available in this article (and supplementary material).

## Supplementary Materials

The following are available online at https://www.mdpi.com/2072-6694/13/2/319/s1, Figure S1: UCB-CD34^+^ cells were isolated and cultured with appropriate cytokines (i.e., SCF+FLt3-L+IL-7+IL15) in the absence (CTR) or in the presence of GSK126 or UNC1999 at the final concentration of 1 mM. (A) Dot plots show the surface staining of indicated markers on CD56^+^ cells developed in the presence of cytokines alone (CTR) after 25 days of culture. Representative experiment out of 10. (B) Dot plots show the analysis of Annexin V/Propidium Iodide staining observed in CD56^+^CD14^−^, CD56^+^CD117^+^ and CD56^+^CD94/NKG2A^+^ cells after 8 and 20 days of culture. Representative experiments out of three. Cells were analyzed after 48h since the EZH1/2 inhibitors were added to the culture. (C) Dot plots show the staining of indicated surface and intranuclear markers expressed by CD56^+^CD14^−^ cells obtained after 25 days of culture in the absence (CTR) or in the presence of GSK126 at 1 μM concentration. Representative experiment out of nine, Figure S2: After 27 days of culture CD14^−^CD56^+^ cells were analyzed for the expression of CD107a and for intra-cytoplasmic expression of Perforin. (A) Dot Plots shows CD107a staining on CD45^+^CD56^+^CD14^−^ cells and on relative LFA-1^+^CD94^+^ and CD94^−^LFA-1^−^ cell subsets after incubation with K562 human leukemic cell lines. A representative experiment out of two performed with UNC1999 (UNC) at 1 μM concentration is shown. (B) Dot plots display the analysis of intra-cytoplasmic Perforin staining in CD56^+^ cells and in CD56^+^LFA-1^+^ cell subset undergone differentiation in the absence (CTR) or in the presence of GSK126 (GSK) or UNC1999 (UNC) at 1 μM concentration. Representative experiment out of three. (C) The histogram shows the percentages of CD56^+^Perforin^+^ cells and CD56^+^LFA-1^+^Perforin^+^ cell subset developed in the absence (CTR) or in the presence of GSK126 (GSK) or UNC1999 (UNC) at 1 μM concentration. The data are represented as the Mean values ± SEM obtained by 4 independent experiments, Figure S3: CD14^−^CD56^+^ cells recovery after 15 days of culture with appropriate cytokines (i.e., SCF+FLt3-L+IL-7+IL15) in the absence (CTR) or in the presence of UNC1999 at the final concentration of 3, 2 and 1 μM. (A) The right panel shows the dot plots staining of indicated surface makers on CD56^+^CD14^−^ cells developed in the absence (CTR) or in the presence of different concentration of UNC1999. Representative experiment out of two. (B) The left panel shows the histogram of absolute cell numbers of total and CD56^+^ cell recovery after 15 days of culture in the absence (CTR) or in the presence of different concentration of UNC1999 (3–2–1 μM). The data are represented as the Mean values ± SEM obtained by 2 independent experiments.
