# Supplementary material for: EZH1/2 Inhibitors Favor ILC3 Development from Human HSPC-CD34+ Cells
Source: Cancers (Basel). 2021 Jan 16;13(2):319. doi: 10.3390/cancers13020319 (PMC7830003; doi:10.3390/cancers13020319)
Supplement: Supplementary file 1 [file cancers-13-00319-s001.zip › cancers-1043385-SI/Figure S1 Damele et al..pdf]

Figure S1

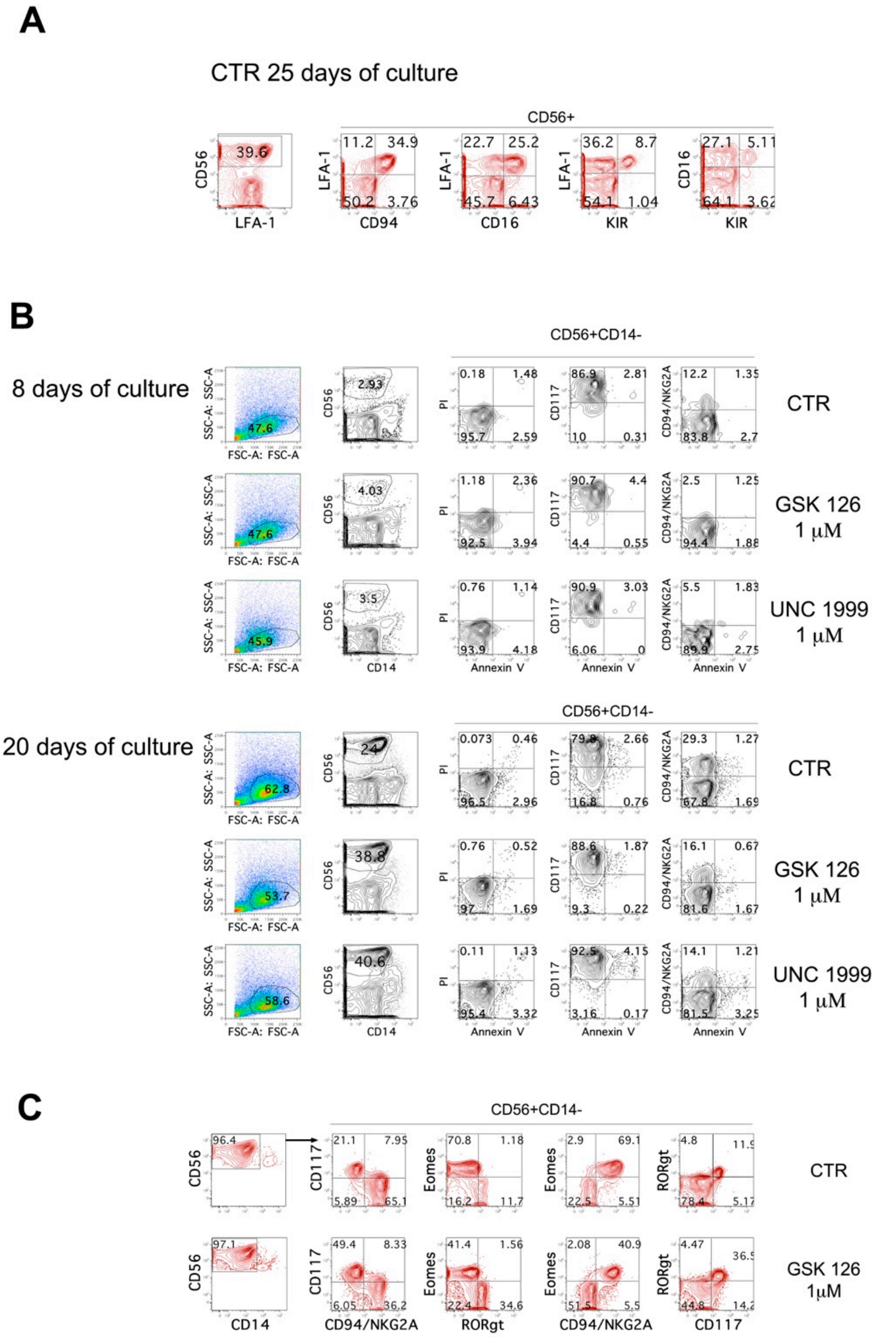

Figure S1. UCB-$CD34^+$ cells were isolated and cultured with appropriate cytokines (i.e. SCF+FLt3-L+IL-7+IL15) in the absence (CTR) or in the presence of GSK126 or UNC1999 at the final concentration of 1μM. A) Dot plots show the surface staining of indicated markers on $CD56^+$ cells developed in the presence of cytokines alone (CTR) after 25 days of culture. Representative experiment out of 10. B) Dot plots show the analysis of Annexin V/Propidium Iodide staining observed in $CD56^+CD14^-$, $CD56^+CD117^+$ and $CD56^+CD94/NKG2A^+$ cells after 8 and 20 days of culture. Representative experiments out of three. Cells were analyzed after 48h since the EZH1/2 inhibitors were added to the culture. C) Dot plots show the staining of indicated surface and intranuclear markers expressed by $CD56^+CD14^-$ cells obtained after 25 days of culture in the absence (CTR) or in the presence of GSK126 at 1 μM concentration. Representative experiment out of nine.
