# Supplementary material for: EZH1/2 Inhibitors Favor ILC3 Development from Human HSPC-CD34+ Cells
Source: Cancers (Basel). 2021 Jan 16;13(2):319. doi: 10.3390/cancers13020319 (PMC7830003; doi:10.3390/cancers13020319)
Supplement: Supplementary file 1 [file cancers-13-00319-s001.zip › cancers-1043385-SI/Figure S2 Damele et al..pdf]

Figure S2

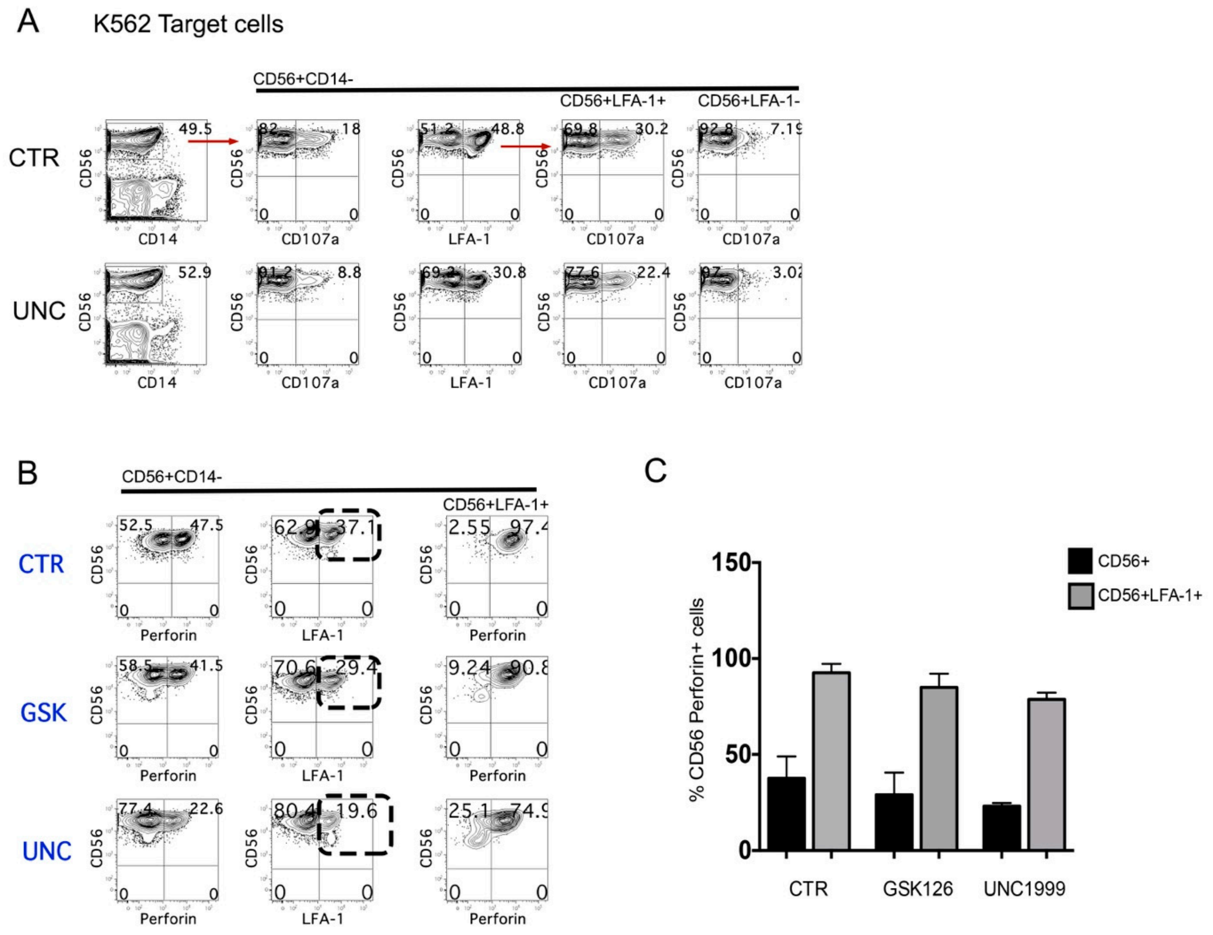

Figure S2. After 27 days of culture CD14⁻CD56⁺ cells were analyzed for the expression of CD107a and for intra-cytoplasmic expression of Perforin. A) Dot Plots shows CD107a staining on CD45⁺CD56⁺CD14⁻ cells and on relative LFA-1⁺CD94⁺ and CD94⁻LFA-1⁻ cell subsets after incubation with K562 human leukemic cell lines. A representative experiment out of two performed with UNC1999 (UNC) at 1 μM concentration is shown. B) Dot plots display the analysis of intra-cytoplasmic Perforin staining in CD56⁺ cells and in CD56⁺LFA-1⁺ cell subset undergone differentiation in the absence (CTR) or in the presence of GSK126 (GSK) or UNC1999 (UNC) at 1 μM concentration. Representative experiment out of three. C) The histogram shows the percentages of CD56⁺Perforin⁺ cells and CD56⁺LFA-1⁺Perforin⁺ cell subset developed in the absence (CTR) or in the presence of GSK126 (GSK) or UNC1999 (UNC) at 1 μM concentration. The data are represented as the Mean values ± SEM obtained by 4 independent experiments.
