# Supplementary material for: EZH1/2 Inhibitors Favor ILC3 Development from Human HSPC-CD34+ Cells
Source: Cancers (Basel). 2021 Jan 16;13(2):319. doi: 10.3390/cancers13020319 (PMC7830003; doi:10.3390/cancers13020319)
Supplement: Supplementary file 1 [file cancers-13-00319-s001.zip › cancers-1043385-SI/Figure S3 Damele et al. .pdf]

Figure S3

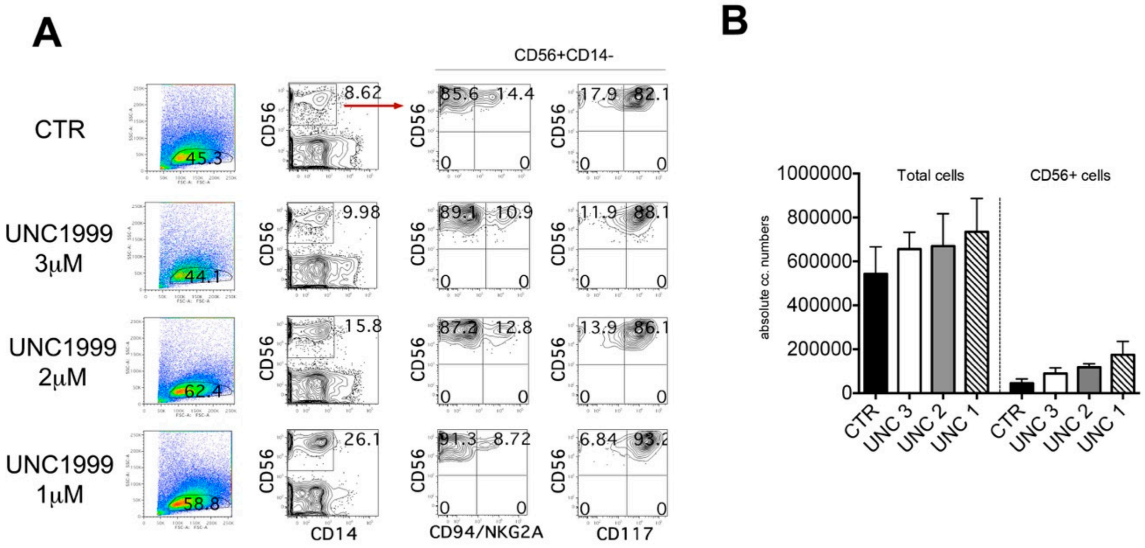

Figure S3. CD14⁻CD56⁺ cells recovery after 15 days of culture with appropriate cytokines (i.e. SCF+FLt3-L+IL-7+IL15) in the absence (CTR) or in the presence of UNC1999 at the final concentration of 3,2 and 1μM. A) The right panel shows the dot plots staining of indicated surface makers on CD56⁺CD14⁻ cells developed in the absence (CTR) or in the presence of different concentration of UNC1999. Representative experiment out of two. B) The left panel shows the histogram of absolute cell numbers of total and CD56⁺ cell recovery after 15 days of culture in the absence (CTR) or in the presence of different concentration of UNC1999 (3-2-1 μM). The data are represented as the Mean values ± SEM obtained by 2 independent experiments.
